# A67 CASE REPORT: A RARE CASE OF MALAKOPLAKIA

**DOI:** 10.1093/jcag/gwae059.067

**Published:** 2025-02-10

**Authors:** S D Bray, J Myette, A Stueck, H Petropolis

**Affiliations:** Division of Digestive Care & Endoscopy, Dalhousie University, Halifax, NS, Canada; Division of Digestive Care & Endoscopy, Dalhousie University, Halifax, NS, Canada; Division of Digestive Care & Endoscopy, Dalhousie University, Halifax, NS, Canada; Division of Digestive Care & Endoscopy, Dalhousie University, Halifax, NS, Canada

## Abstract

**Background:**

Malakoplakia is a rare chronic granulomatous inflammatory condition first described in the literature in 1902^1^. It is thought to be a result of impaired macrophages ability to completely digest and kill bacteria, resulting in phagocytosed bacteria accumulating in macrophages calcifying overtime. Malakoplakia can affect all organs of the body and is four-times more likely to affect females^2^. It most commonly occurs within the genitourinary tract with the gastrointestinal tract being the second most common site.

**Aims:**

The literature regarding malakoplakia of the rectum is limited and rare^3^.

**Methods:**

This 39-year-old immunosuppressed male with a history of three renal transplants receiving tacrolimus and chronic prednisone found to have rectal malakoplakia. He was referred to gastroenterology for symptoms of constipation and iron deficiency anemia. A colonoscopy was completed which demonstrated 4-rectal polyps, each at an estimated size of 5 mm which were removed by cold snare.

**Results:**

The rectal polyps were sent for histological analysis and demonstrated expansion of cells into the lamina propria. Cells were mostly epithelioid histiocytes with some admixed neutrophils, lymphocytes, and plasma cells, but infiltration did not destroy the mucosal architecture or crypts. Some of the histiocytes contain pale, round intracytoplasmic structures with concentric layering, consistent with Michaelis-Gutmann bodies. CD68 confirmed infiltrating cells were macrophages, confirming a diagnosis of rectal malakoplakia (Figure 1).

**Conclusions:**

He was seen in follow-up in Gastroenterology clinic and his symptoms of constipation, did not appear to be contributory towards this incidental finding of malakoplakia. Furthermore, infectious disease was involved in this patient’s care for consideration of antimicrobial therapy, but a decision was made to treat conservatively and monitor for any new gastrointestinal symptoms. This patient is followed by gastroenterology and booked for repeat colonoscopy in 6-months for follow-up to determine if these lesions can be eradicated through polypectomy.

1. Liu, Xiangyu, et al. “Rectal malakoplakia mimicking advanced rectal cancer: A case report.” *Heliyon* 9.10 (2023).

2. Wang, Zhenting, and Jiannan Ren. “Clinical analysis of renal failure caused by malakoplakia: a case report and literature review.” *Frontiers in Medicine* 9 (2022): 770731.

3. Achufusi, T. G., Jessamy, K., Chebaya, P., & Rawlins, S. (2020). Rectal malakoplakia. *Proceedings (Baylor University. Medical Center)*, *33*(3), 389–390.

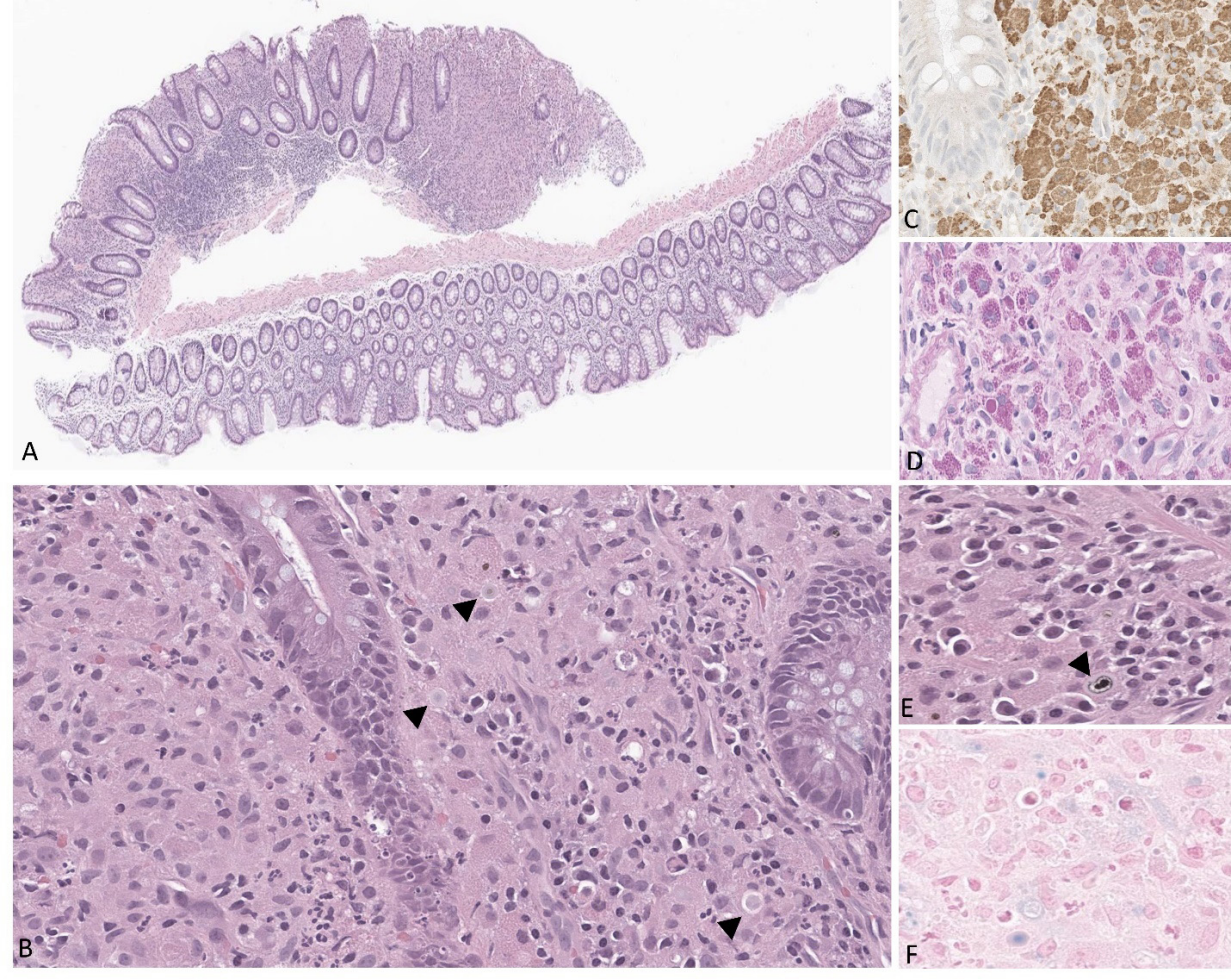

**Funding Agencies:**

